# Targeting Specific Checkpoints in the Management of SARS-CoV-2 Induced Cytokine Storm

**DOI:** 10.3390/life12040478

**Published:** 2022-03-25

**Authors:** Abdullahi Rabiu Abubakar, Rahnuma Ahmad, Adekunle Babajide Rowaiye, Sayeeda Rahman, Katia Iskandar, Siddhartha Dutta, Angus Nnamdi Oli, Sameer Dhingra, Maryam Abba Tor, Ayukafangha Etando, Santosh Kumar, Mohammed Irfan, Marshall Gowere, Kona Chowdhury, Farhana Akter, Dilshad Jahan, Natalie Schellack, Mainul Haque

**Affiliations:** 1 Department of Pharmacology and Therapeutics, Faculty of Pharmaceutical Sciences, Bayero University, PMB 3452, Kano 700233, Nigeria; unisza7@gmail.com; 2 Department of Physiology, Medical College for Women and Hospital, Dhaka 1230, Bangladesh; rahnuma.ahmad@gmail.com; 3 National Biotechnology Development Agency, Abuja 09004, Nigeria; adekunlerowaiye@gmail.com; 4 School of Medicine, American University of Integrative Sciences, Bridgetown BB11114, Barbados; srahman@auis.edu; 5 Department of Pharmaceutical Sciences, Faculty of Pharmacy, Lebanese University, Beirut P.O. Box 6573/14, Lebanon; katia_iskandar@hotmail.com; 6 Department of Pharmacology, All India Institute of Medical Sciences, Rajkot 360001, Gujrat, India; siddhartha.dutta87@gmail.com; 7 Department of Pharmaceutical Microbiology and Biotechnology, Faculty of Pharmaceutical Sciences, Nnamdi Azikiwe University, PMB 5025, Awka 420110, Nigeria; an.oli@unizik.edu.ng; 8 Department of Pharmacy Practice, National Institute of Pharmaceutical Education and Research (NIPER), Hajipur 844102, Bihar, India; sameerdhingra78@gmail.com; 9 Department of Health and Biosciences, University of East London, University Way, London E16 2RD, UK; u1862562@uel.ac.uk; 10 Department of Medical Laboratory Sciences, Faculty of Health Sciences, Eswatini Medical Christian University, P.O. Box A624 Swazi Plaza Mbabane, Mbabane H101, Hhohho, Eswatini; etta5013@gmail.com; 11 Department of Periodontology and Implantology, Karnavati School of Dentistry, Karnavati University, 907/A, Adalaj Uvarsad Road, Gandhinagar 382422, Gujarat, India; santosh@ksd.ac.in; 12 Department of Forensics, Federal University of Pelotas, R. Gomes Carneiro, 1-Centro, Pelotas 96010-610, RS, Brazil; irfan_dentart@yahoo.com; 13 Department of Pharmacology, Faculty of Health Sciences, Basic Medical Sciences Building, Prinshof Campus, University of Pretoria, Arcadia 0083, South Africa; u18064397@tuks.co.za (M.G.); natalie.schellack@up.ac.za (N.S.); 14 Department of Paediatrics, Gonoshasthaya Samaj Vittik Medical College and Hospital, Dhaka 1344, Bangladesh; konaonu96@gmail.com; 15 Department of Endocrinology, Chittagong Medical College, Chattogram 4203, Bangladesh; fakter36@gmail.com; 16 Department of Hematology, Asgar Ali Hospital, 111/1/A Distillery Road, Gandaria Beside Dhupkhola, Dhaka 1204, Bangladesh; dilshad@asgaralihospital.com; 17 Unit of Pharmacology, Faculty of Medicine and Defense Health, Universiti Pertahanan Nasional Malaysia (National Defense University of Malaysia), Kem Perdana Sungai Besi, Kuala Lumpur 57000, Malaysia

**Keywords:** cytokine storm, SARS-CoV-2, COVID-19, pathogenesis, immune response, interleukins, hyperinflammation

## Abstract

COVID-19-infected patients require an intact immune system to suppress viral replication and prevent complications. However, the complications of SARS-CoV-2 infection that led to death were linked to the overproduction of proinflammatory cytokines known as cytokine storm syndrome. This article reported the various checkpoints targeted to manage the SARS-CoV-2-induced cytokine storm. The literature search was carried out using PubMed, Embase, MEDLINE, and China National Knowledge Infrastructure (CNKI) databases. Journal articles that discussed SARS-CoV-2 infection and cytokine storm were retrieved and appraised. Specific checkpoints identified in managing SARS-CoV-2 induced cytokine storm include a decrease in the level of Nod-Like Receptor 3 (NLRP3) inflammasome where drugs such as quercetin and anakinra were effective. Janus kinase-2 and signal transducer and activator of transcription-1 (JAK2/STAT1) signaling pathways were blocked by medicines such as tocilizumab, baricitinib, and quercetin. In addition, inhibition of interleukin (IL)-6 with dexamethasone, tocilizumab, and sarilumab effectively treats cytokine storm and significantly reduces mortality caused by COVID-19. Blockade of IL-1 with drugs such as canakinumab and anakinra, and inhibition of Bruton tyrosine kinase (BTK) with zanubrutinib and ibrutinib was also beneficial. These agents' overall mechanisms of action involve a decrease in circulating proinflammatory chemokines and cytokines and or blockade of their receptors. Consequently, the actions of these drugs significantly improve respiration and raise lymphocyte count and PaO_2_/FiO_2_ ratio. Targeting cytokine storms' pathogenesis genetic and molecular apparatus will substantially enhance lung function and reduce mortality due to the COVID-19 pandemic.

## 1. Introduction 

The Severe Acute Respiratory Syndrome Coronavirus 2 (SARS-CoV-2) has led to an unprecedented global health crisis called COVID-19. In view of the severity and wide spread of this condition, the World Health Organization (WHO) declared it a pandemic on 11 March 2020 [1]. As reported by WHO on 16 February 2022, there were 412,351,279 cases and 5,821,004 deaths across the globe [2]. The presentation of the disease varies from mild to severe forms of fever, cough, and myalgia. Moderate symptom presentation may require hospitalization; for severe cases the prognosis is worse, and they mainly require intensive care [3,4,5]. Patients with comorbid conditions usually had more complicated scenarios associated with a worse prognosis. Complications of COVID-19 include acute respiratory distress syndrome (ARDS), altered coagulation profile, and eventually multiorgan failure [3,6,7,8]. Other common symptoms of COVID-19 include running nose, headache, diarrhea, conjunctivitis, and sore throat [9,10]. SARS-CoV-2 gets into the host cell by attaching to the angiotensin-converting enzyme 2 (ACE2) receptors widely located across various tissues and immune cells [11,12]. Evidence suggested that elevated levels of pro-inflammatory cytokines such as interleukins (IL)-1, IL-2, IL-6, IL-7, tumor necrosis factor-α (TNF-α), and interferon-gamma (INF-γ), as well as the activation of immune cells, were associated with poor prognosis [13,14,15,16,17]. Consequently, the high levels of cytokines culminated into a hyperinflammatory syndrome, also termed a cytokine storm [13,15,18]. 

The swift spread of COVID-19 infection coupled with associated morbidity and mortality prompted the government to impose lockdowns that further escalated the financial burden of the disease [19,20]. There was a dire need for therapeutic agents to contain the pandemic. However, in the absence of definitive treatment, the existing medicines and complementary and alternative therapies were being repurposed to prevent and treat COVID-19 cases [13,21,22,23]. Unfortunately, most of the repurposed therapies failed to benefit most of the clinical trials conducted across the globe [24,25,26]. Later, with the advent of COVID-19 vaccines from various multinational pharmaceutical industries, there was a mass vaccination to immunize people and contain the spread of the virus [27,28,29].

It is noteworthy that even after almost two years, the COVID-19 pandemic is yet to be contained, and few countries are even experiencing a rising trend in the number of new cases [30]. Cytokine storm is one of the critical complications of COVID-19 infection leading to death. Therefore, we need therapeutic agents that can target the critical checkpoints involved in the pathogenesis of the cytokine storm to minimize the morbidity and mortality associated with this viral disease. 

## 2. Objectives of the Study

This article reviews the most recent information about pathogenesis, molecular interplay, and various checkpoints targeted to manage the SARS-CoV-2-induced cytokine storm. 

## 3. Materials and Methods

Information was searched using electronic databases including PubMed, Embase, MEDLINE, and China National Knowledge Infrastructure (CNKI) using Google Scholar as the search engine. The databases were first searched individually for the relevant articles. The search terms used include ‘Pathogenesis of SARS-CoV-2’, ‘Molecular Mechanism’, ‘Cytokine Storm’, ‘Hyperinflammatory Syndrome’, ‘COVID-19-Induced Thromboinflammation’, ‘SARS-CoV-2 Oxidative Stress’, ‘Immune Response’, ‘Diagnosis of SARS-CoV-2-Induced Hyperinflammation’. This review included pre-printed articles where necessary because research on the COVID-19 pandemic is a new area of study. The quality of the articles reviewed was not scrutinized using Newcastle Ottawa Scale. Journal articles that discuss SARS-CoV-2 infection and cytokine storm and written in the English language were finally selected and reviewed.

## 4. Pathogenesis of SARS-CoV-2 Infection

**Figure 1:**
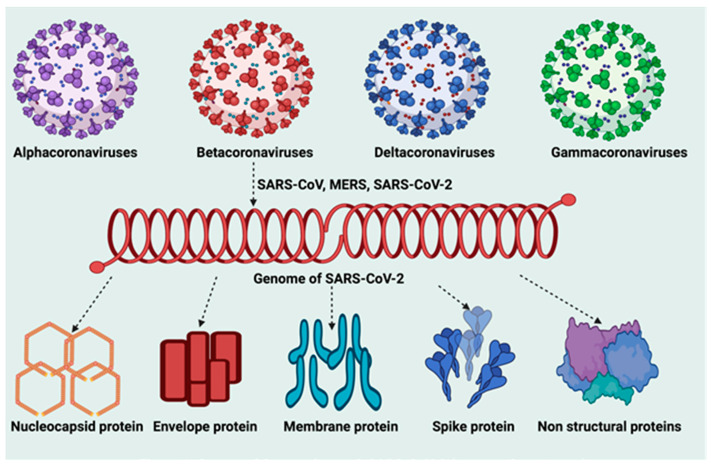
Genera of coronaviruses and SARS-CoV-2 (genome and proteome).

**Figure 2:**
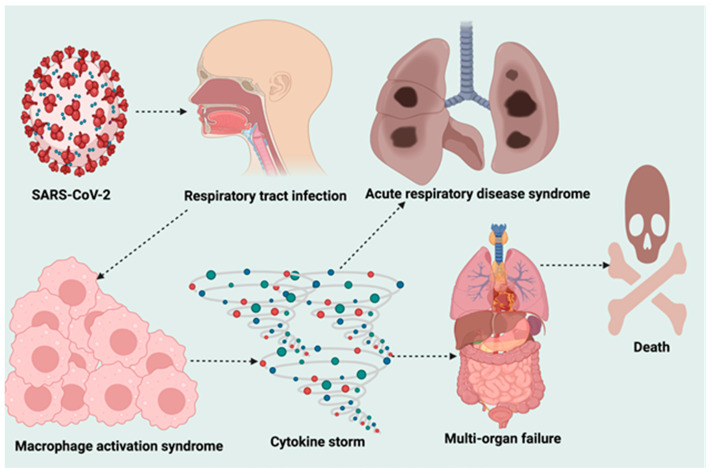
Molecular pathogenesis of SARS-CoV-2-induced cytokine storm.

**Figure 3:**
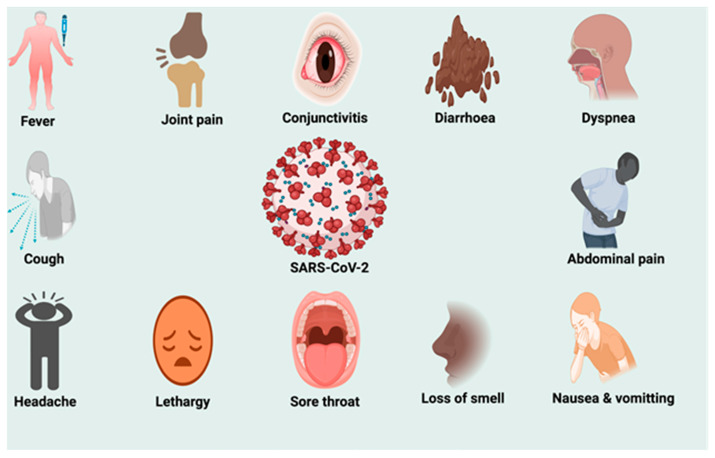
Symptoms of SARS-CoV-2 infection.

Coronaviruses are a large family of enveloped, positive-sense, and single-stranded RNA viruses. They are divided into four genera: α, β, δ, and γ coronaviruses (Figure 1). The α and β coronaviruses are known to infect humans [31]. The current SARS-CoV-2 belongs to the β-coronaviruses in the same genus as the earlier SARS-CoV-1 [32,33,34] and the Middle East respiratory syndrome (MERS) virus [35]. SARS-CoV-2 closely resembles two bat coronaviruses, according to full-genome sequencing and phylogenetic analyses [36,37]. SARS-CoV-2 has a genome size between 26.2 and 31.7 kb [38]. It consists of Structural and Non-Structural Proteins (NSPs) that are necessary for propagation of its pathophysiological (Figure 2) processes [35]. The incubation period for SARS-CoV-2 is 1–14 days, and when in close contact with infected persons, it is transmitted predominantly through saliva and respiratory droplets from coughing, sneezing, or talking [39,40]. The virus has also been found in COVID-19 patients' feces and urine, implying a fecal–oral transmission route [41]. The primary predilection site of COVID-19 is the upper and lower respiratory tracts. The most common symptoms include fever, cough, lethargy, anorexia, dyspnea, sore throat, headache, conjunctivitis, sputum production, muscle, and joint pains, smell and taste loss, diarrhea, nausea, vomiting, and gastrointestinal disturbances (Figure 3) [39,42,43].

## 5. Molecular Anonymity of SARS-CoV-2 Infection

### 5.1. Molecular Structure of SARS-CoV-2

**Figure 4:**
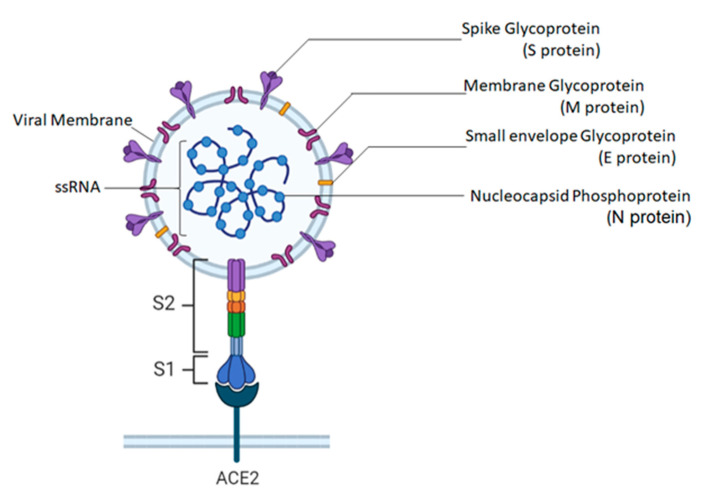
Molecular structure of SARS-CoV-2.

SARS-CoV-2 is a spherical and enveloped virus with specific surface projections [44]. Through microscopic view, the structure of the SARS-CoV-2 virus appears (Figure 4) like a crown due to its series of protein spikes on its surface that gives rise to its name corona, a Latin word meaning ‘crown’. Corona viral particles are pleomorphic implying that they do not have a defined structure [45]. This is revealed based on the outcome of Cryo-electron tomography [45,46]. Coronaviruses generally contain non-segmented, single-stranded, positive-sense ribonucleic acid (ssRNA+) as the genome, wrapped up in helical nucleocapsid [45]. Among the RNA viruses, coronaviruses have the largest genome size. The genome size of SARS-CoV-2 is about 30 kb [47]. Two-thirds of the 5′ end of this genome encodes for gene 1 proteins that control the synthesis of viral RNA, and one-third of the 3′ end is responsible for encoding all the structural and accessory proteins [48]. Four structural proteins are similar to all coronaviruses: S (spike), E (envelope), M (membrane), and N (nuclear capsid) proteins (Figure 1). Two-thirds of the SARS-CoV-2 genome consists of replicase genes processing polyproteins, pp1a and pp1ab, which are later converted into 16 nonstructural proteins through proteolytic cleavage [49,50,51]. 

The spike protein is critical for viral entry. It is the most abundant protein outside of the viral particle used to invade the host’s cell membrane [52]. It contains a receptor-binding domain that recognizes the angiotensin-converting enzyme receptor 2 (ACE2) expressed in the lungs, heart, kidneys, and intestines [53]. The spike protein of SARS-CoV-2 binds to the ACE2 receptor with 20 times greater affinity than other SARS viruses [54,55]. This could be one of the explanations for spreading so quickly [56]. The penetration occurs through the endocytosis process [57], and the binding to the ACE2 receptors provides a proteolytic cleavage event, carried out by a cellular protease called TMPRSS2 [57]. The spike protein is a class1 fusion protein [58]; it has two functional subunits, S1 that binds to the host cell receptor, and S2, which mediates the fusion of the viral and cellular membranes. [59]. The C-terminal domain contacts the nucleocapsid protein and is vital for the morphogenesis phase of the viral life cycle when the virions are formed [60]. Depending on the virus, either NTD or CTD can serve as the receptor-binding domain (RBD) [44]. Because of the critical role of s-protein in binding to the host’s cells, it could be targeted in designing vaccinations and medical treatments for COVID-19 [61]. Mutations of this spike protein are likely to increase viral infectivity and transmissibility and reduce the efficacy of drugs and vaccines [62]. For instance, spike protein mutations in the RBD N501Y lead to the emergence of the α variant (B.1.1.7) [63]. This mutation had increased the affinity of the virus to the ACE2 receptors and has amplified the viral replication in the lungs [63].

Coronaviruses have another minor envelope protein called E necessary to form viral particles at the end of the viral life cycle [64]. It is integral for the assembly and release of the virus from host cells. It is localized to the site of intracellular trafficking during viral replication, specifically at the endoplasmic reticulum and the Golgi apparatus [65]. The virus has an additional glycoprotein membrane on its layer called the matrix protein. This transmembrane protein has a significant C-terminal domain that contacts the N protein [64,66].

The M protein is the most abundant on the viral surface and defines the shape of the viral envelope. It is considered the central organizer for coronavirus assembly and interactions with the other structural proteins [67]. The viruses’ outer layer is derived from the hosts’ cell membrane. As viruses cannot make their lipids, they use the host’s lipids for replication and morphogenesis [68]. This protein shell encloses the genetic material of the virus. It has the helical nucleocapsid or N protein inside this capsid, carrying its genetic information within the single-stranded viral RNA [69]. The N protein appears to be multifunctional. In particular, it essentially inhibits many host cells' defense mechanisms and assists the viral RNA replication, creating new viral particles [66]. The M and E proteins play a critical role in turning the host cell apparatus into workshops where the virus and host cells work together to make new viral particles [59]. 

### 5.2. Viral Replication of SARS-CoV-2

Once the coronavirus enters the host’s respiratory tract, it effectively binds, using its spike protein, to ACE2 receptors present in the type II pneumocyte [68,69,70,71]. The virus membrane fuses with the host cell membrane facilitating the entry of viral ssRNA into the host cell cytoplasm [64]. Immediately, the host ribosome confuses viral RNA with the host’s mRNA and starts translating the viral ssRNA into specific protein molecules [64]. The first to be read from the 5′ region of the positive strand of viral RNA is the Leader Sequence, enabling proper alignment with the host ribosome. This follows the 5′ untranslated region, i.e., UTR Sequence, that regulates viral translation [72]. The first essential protein to be produced is the RNA-dependent RNA polymerase, responsible for replicating viral RNA. Three critical structural proteins are translated of which, first, is S-Protein in its non-glycosylated form.

The coronavirus (CoV) envelope (E) protein is a microscopic, essential membrane protein. This E protein has an out-and-out requirement in multiple features of the virus' life cycle; those include assembly, budding, envelope formation, and pathogenesis [64]. Thereby, this structural protein is translated as Membrane Protein (M), which determines the circular-curved shape of the virus [64,73,74]. Consequently, these proteins are transcribed and translated in the rough endoplasmic reticulum (RER) [75]. Here, all proteins attain their partial maturity and then they are transported to the Golgi apparatus through vesicular transport [75,76]. On maturation, these proteins assemble and polymerize to form a nucleocapsid and stay inside the Golgi body until they receive the replicated viral RNA [77]. It is the only protein found inside the core of the viral structure. The ribosome now slides to the 3′ untranslated region (UTR), which contains a pseudoknot structure essential for viral genome replication [78]. 

The viral RNA replicates in the presence of enzyme replicase. As a rule, the polymerases read the template strand from the 3′ to 5′ position and synthesize the complementary strand in the antiparallel direction from 5′ to 3′ [75]. For the illustration depicted here, the first cycle of positive RNA strand replication will yield its complementary strand, a negative RNA strand. The negative RNA strand is used as a template to produce more positive strands [75]. The positive strands formed after replication will bind with the nucleocapsid protein and are condensed, then transported to the Golgi apparatus and packed into the viral particle [51]. The matured viral particles escape from the Golgi apparatus entrapped inside vesicles. The vesicles containing the viral particles travel to the periphery of the host cell, facilitating the exocytosis of the viral particles [77,78]. These viral particles can now be transmitted to other human hosts [75]. 

### 5.3. Molecular Components of Cytokine Storm (CS)

Cytokines are small soluble molecules that serve as messengers for the immune system; they are signaling molecules that consist of various proteins and glycoproteins [79]. Cytokines regulate the host’s immune responses to infection, inflammation, and trauma [80]. They are produced by various immune cells, such as neutrophils, basophils, eosinophils, mast cells, dendritic cells, monocytes, macrophages, B-cells, and T-cells. The cytokines serve as intercellular mediators by binding to specific receptors called cytokine receptors on the surface of cells. Cytokines have a very high affinity for the cytokine receptors making them effective even at low concentrations [81]. They usually have a short half-life and act over short distances [82,83].

As the cytokines enter the bloodstream, they provide a systemic effect. These change cell activities by altering the functions of cellular proteins or by changing the expression of specific genes [84]. Cytokines play important roles ranging from boosting immunity to supporting the growth, development, maturation, activation, and lifespan of immune cells [84]. Another set of molecules is chemokines, which coordinate white blood cells' movements in the body. Chemokines mobilize both granulocytes (neutrophils) and agranulocytes (monocytes, macrophages, and lymphocytes) to the site of infection [85]. 

The structural groups of cytokines comprise IL, Tumor Necrosis Factor (TNF), interferons (INF), and Colony Stimulating Factors (CSF). Leukocytes produce interleukins that also action other leukocytes [86]. They play a vital role in the body’s immune response. Interleukins may be pro-inflammatory markers whose release worsens the disease conditions (e.g., IL-1, IL-1β, IL-6, IL-8) or anti-inflammatory markers that reduce inflammation and promote healing (e.g., IL-4, IL-10, IL-13). Cytokine storm may also result from decreased neutrophil and monocyte function within the systemic circulation [87,88,89].

Mast cells, macrophages, and T-cells produce the tumor necrosis factor (TNF), which plays a significant role in immune cell activation, differentiation, growth, and death [90]. TNF is the major pro-inflammatory cytokine that activates cytotoxic T-cells during infection and inflammation. Therefore, the blockage of the TNF-α can be targeted to treat autoimmune diseases and inflammatory disorders [90]. 

The two most crucial interferons (IFNs) are produced by virtually all cells; nevertheless; fibroblast and monocytes remain as a significant contributor, and often interfere with viral replication [91]. Twenty different interferons have been identified in humans so far. The main interferons are type 1 and type 2. Type 1 (IFN-α and IFN-ꞵ) are produced by fibroblast and monocytes. The type 2 interferon plays a role in many immune responses and increases the phagocytic activity of macrophages [92]. The anti-viral function is generated when a viral infection attacks a cell. The infected cell usually releases interferons. The interferons then bind with the uninfected neighboring cells and produce antiviral proteins, degrading viral RNA and inhibiting protein synthesis [93]. Type 1 interferons can treat viral infections such as hepatitis C [94].

One more cause of cytokine storms is Colony Stimulating Factors (CSF). These essentially act on stem cells in the bone marrow to stimulate growth and differentiation into specific cells [95,96,97]. Types of CSF include: the Monocyte Colony-Stimulating Factor (M-CSF), which influences the growth of monocytes; Granulocyte-Macrophage Colony-Stimulating Factor (GM-CSF), which helps with the growth and differentiation of dendritic cells; Granulocyte Colony-Stimulating Factor (G-CSF), which supports the differentiation and development of neutrophils [95]. Overall, the CSF plays a significant role in the growth of immune cells, alveolar macrophages homeostasis, lung inflammation, and autoimmune diseases. It also activates T-cells and indirectly causes the release of inflammatory mediators leading to cytokine syndrome [95].

### 5.4. Cytokine Storm Molecular Mechanism in SARS-CoV-2

Cytokine storm (CS) is an exaggerated immune response [98]. In any infection, an immune response is necessary to combat the pathogen. There is a rise in immune response, which lowers over time and eventually resolves. However, in the case of COVID-19, the immune response is dysregulated [88,99]. Fourteen days after the SARS-CoV-2 infection, the immune system gets to ramp up more than it should [98]. This is associated with severe morbidity and mortality [98]. It occurs as a result of the release of many pro-inflammatory cytokines.

Generally, viral cells are replicating rapidly, making thousands of copies that eventually burst out of the host cell. Due to the damage caused to these cells, cytokines and inflammatory markers are released. Subsequently, the virus as an antigen comes in contact with the host’s antibody-containing cells which stimulates B- and T-cell receptors with viral antigens, leading to Ig production [100,101]. Consequently, a series of things happen here. (i) Firstly, production of IgM and IgG antibodies, (ii) this is followed by stimulation of the humoral and cellular immunity mediated by the virus-specific B-cell and T-cells, (iii) release of antibodies and later pro-inflammatory markers that produce the cytokine storm (CS) [98,102]. The CS leads to a powerful attack by the immune system on the body. This typically begins in the lungs and then spreads to the rest of the body [98,102].

**Figure 5:**
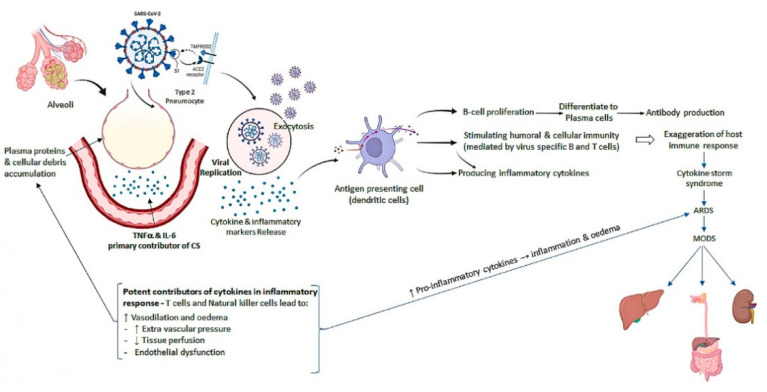
Progression of cytokine storm in COVID-19. CS—cytokine storm; ARDS—acute respiratory distress syndrome; MODS—multiple organ dysfunction syndromes.

The body’s T-cells and natural killer (NK) cells trigger release of cytokines, leading to the inflammatory response. In addition, they cause vasodilation and edema, which ultimately (a) increase the extravascular pressure, (b) decrease tissue perfusion, (c) endothelial dysfunction, and (d) compromise the integrity of endothelial cells [103]. The CS in the alveoli can lead to acute lung injury (Figure 5), which can progress into ARDS. Eventually, fibrosis still leaves some progressive dysfunction [98,102]. Therefore, some of these long-term effects may be experienced by the patients over an extended period. The CS can happen systemically and may affect other organ systems in the body leading to systemic clinical presentations known as multi-organ failures. The CS may affect the renal system, hepatic system, GI, cardiovascular, and CNS [104,105,106,107].

## 6. SARS-CoV-2 Viral Load and Cytokine Storm 

Among the human coronaviruses, MERS-CoV, SARS-CoV-1, and SARS-CoV-2 replicate in the lower respiratory tract and result in lethal pneumonia in many instances. Once the SARS-CoV-2 virus invades the human body, the viral load attains a peak value in 5–6 days. Consequently, the signs and symptoms of COVID-19 develop within 14 days of infection in about 97.5% of individuals [108,109,110,111,112,113]. A study that compared the behavior of SARS-CoV-1 and SARS-CoV-2 in pulmonary tissue revealed a rapid viral replication by both viruses. An ex vivo experiment in human lung tissue reported an efficient invasion and replication by the SARS-CoV-2 virus in pulmonary tissues. Notably, the SARS-CoV-2 virus caused upregulation of all five inflammation factors, including IL6, CCL2, CXCL 10, CXCL5, and CXCL1 [114]. Another ex vivo study was conducted to compare virus replication and tropism among SARS-CoV-1, SARS-CoV-2, MERS, and H1N1 viruses using human bronchus and lung samples. During the experiment, samples of these viruses were obtained from the human conjunctival epithelium and human colorectal adenocarcinoma cell lines to observe extra pulmonary infection's viability [115]. The result showed that SARS-CoV-2 virus infected the ciliated and mucus-secreting cells of the epithelium of bronchi, type 1 pneumocytes of the lung, and mucosa of the conjunctiva. There was a higher SARS-CoV-2 replication in the bronchi than SARS and MERS [116].

One more study concluded that the relationship between SARS-CoV-2 viral load and COVID-19 disease progression. The viral load was quantified from COVID-19 patients with mild, moderate, and severe cases [117]. This study revealed that of patients diagnosed with COVID-19 who had SARS-CoV-2 plasma RNA, 27% were hospitalized, and 13% of those were treated as outpatients. Hospitalized patients had high levels of lymphocyte counts, inflammatory biomarkers, poor respiratory-related clinical outcomes, and increased mortality risk [117]. In SARS-CoV-2 infection, a higher viral load and amplified immune response results in a cytokine storm. The appearance of a cytokine storm provokes ARDS and multiple organ failure leading to death [118,119,120,121]. In COVID-19-infected patients, there was an activation of alveolar epithelial cells, macrophages, and monocytes by toll-like receptors with the production of a large number of cytokines and attraction of immune cells, causing extensive pulmonary hyperinflammation [7,106,122,123,124,125,126,127]. The IL 6 is responsible for the aggravation of intravascular coagulation leading to injury and multiple organ damage [128,129,130,131,132]. 

## 7. Genetic and Molecular Susceptibility to SARS-CoV-2 Infection 

**Figure 6:**
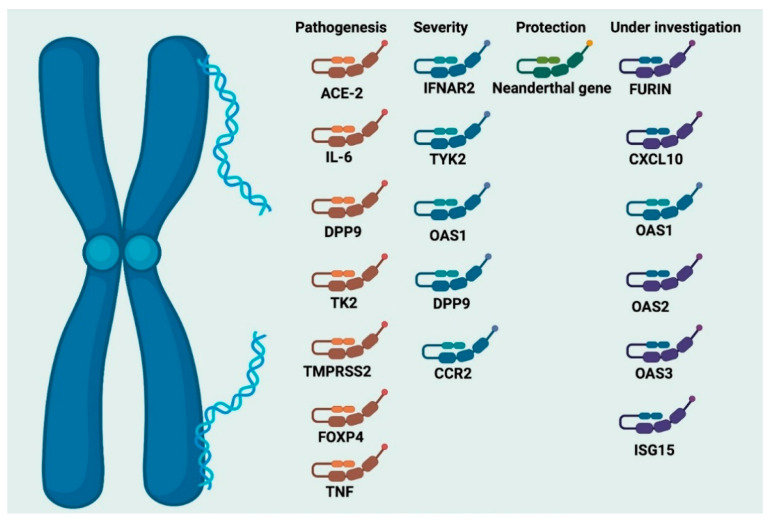
Genes associated with SARS-CoV-2 infection.

Genetic epidemiology has provided valid proof that variations/mutations in the human genomes (Figure 6) play some roles in susceptibility to infectious disease [133]. The dominant view opines that rare, ‘conventional’, monogenic primary immune compromise makes humans prone to a myriad of diseases that result from pathogen invasion, growth, and survival [133,134].

Common communicable diseases involve polygenic inheritance [133,134]. Studies have highlighted relevant genes predisposing a family or a population to some communicable diseases through family-based and population-based approaches [134,135,136,137,138,139]. 

Several methods and approaches have been utilized to identify and map out some genes designated as susceptible or resistant loci for infectious diseases. Such strategies include genome scanning of multi-case families, mouse genetics, screening of likely candidate genes, and genome-wide association studies [140,141,142,143]. In humans, six genes linked to infectious disease susceptibility have been identified [132,144]. Broad knowledge of the impact of human genetics on susceptibility to infections will explain infectious disease pathogenesis, revealing possible drug therapy and vaccination [145].

### 7.1. The Major Genetic Risk Factors for SARS-CoV-2 Infection

The challenge of emerging and re-emerging infections, global antimicrobial resistance, the length of time needed to bring a new drug product into the market, the growing need for personalized medicine, and the genetic differences within and between populations necessitate the inquiry into the relationship between human genetics and infections [145]. 

Five essential genes (IFNAR2, TYK2, OAS1, DPP9, and CCR2) (Table 1) have been linked to the most severe forms of COVID-19 disease, suggesting possible drug targets and vaccine epitopes. These genes are involved in lung inflammation and antiviral immunity [146,147,148]. Augmentation of the INFAR2 gene activity was found to induce protection against COVID-19 [149,150]. 

### 7.2. Genetic Fingerprints for Critical Illness in COVID-19

The containment of COVID-19 involves gaining insight into the genetics of SARS-CoV-2 and the range of the diseases brought about by the infection. The genetic makeup contributes to the progression and prognosis of viral infections [151]. The Major Histocompatibility Complex (MHC) Class I molecules play critical functions in initiating, developing, and expressing specific immune responses against viral infections and cancers [151]. Other genes commonly implicated in coronavirus disease 2019 include ACE2, IL6, DPP9, TYK2, TMPRSS2, FOXP4, and TNF, while the emerging genes consist of FURIN, CXCL10, OAS1, OAS2, OAS3, and ISG15 (Table 1) [152]. Inhibiting some of these genes could be a potential treatment strategy for COVID-19 [153]. Additionally, the differences in the genes’ allele affinities for SARS-CoV-2 peptides are associated with infection severity and mortality [154,155]. 

**Table 1:** Genes, their polymorphism, and specific role in the pathogenesis of COVID-19 infection.

GENES	Full Name	Polymorphism	Specific Role	Reference
IFNAR2	Interferon alpha and beta receptor 2	NM_000874:exon9:c.C966A:p.Y322X	Severe COVID-19-risk	Schmiedel et al., 2021 [156]
IFITM3	Interferon-induced transmembrane protein 3	rs12252-C/C	It is a risk factor for developing severe influenza	Kaidashev et al., 2021. [157]
OAS1	Oligoadenylate synthase 1	rs2057778	Increased risk of infection	Schmiedel et al., 2021 [156]
CCR2	Chemokine receptor	rs11385942-GA	Respiratory failure	Anastassopoulou et al., 2020 [158]
ACE2	Angiotensin Converting Enzyme 2	p.Arg514-Gly	Increase in pulmonary and cardiovascular complications in the African American population	Anastassopoulou et al., 2020 [158]
IL6	Interleukin-6	rs180079	Associated with the increase in susceptibility and severity	Kaidashev et al., 2021. [157]
TMPRSS2	Transmembrane serine protease 2	rs12329760	Increased susceptibility to disease	Anastassopoulou et al., 2020 [158]
HLA	Human leukocyte antigen (HLA) system	HLA-B*46:01	Exhibit the lowest binding cap	Pollitt et al., 2020. [159]
TNF	Tumor necrosis factor	rs1800629	Increase in pulmonary complications	Fishchuk et al., 2021. [160]
FURIN	Furin	rs16944971	Promotes entry of the virus into the cell	Kucher et al., 2021 [161]
CXCL10	Chemokine ligand 10	rs11385942-GA	Respiratory failure	Anastassopoulou et al., 2020 [158]

### 7.3. The Neanderthal Gene Variant and Coronavirus Disease-19 

Zeberg and Pääbo (2021), in their recent study, showed the presence of a protective Neandertal haplotype [162]. This is a set of genetic determinants located on a region at chromosome number 12 and is not associated with patients that require intensive care when infected with SARS-CoV-2. This region was inherited from Homo sapiens Neanderthalensis. Typically, this region is responsible for producing proteins capable of activating the receptor needed for infections with RNA viruses [162]. The protective Neanderthal haplotype is different from the risk haplotype (Neanderthal gene variation on chromosome number 3, which substantially heightens the likelihood of fatal COVID-19) because the former confers a highly reduced effect of the SARS-CoV-2 infection and has prevalence in all regions of the globe but is low in Africa [163,164,165].

### 7.4. Resistance to Coronavirus Disease-19 

SARS-CoV-2 infections have shown variable prognoses among patients. The prognosis varies from symptomless infections to potentially fatal diseases. The proportions of the human population inherently resistant to SARS-CoV-2 infection, together with the genetic and immunological determinants of resistance, are largely unclear. However, some candidate genes have been suggested to be possibly linked to natural human resistance to SARS-CoV-2 infection [166,167,168].

Inherited errors of Type I interferons-self-immunoglobulins contribute up to 20% of severe COVID-19 cases seen among SARS-CoV-2 infection. It is necessary to identify, recruit, and genetically analyze individuals with inborn resistance to SARS-CoV-2 infection [154]. This group of individuals is likely to become an antigenic source to provide more reliable vaccines and other immunotherapies for the global containment of COVID-19 [154].

## 8. SARS-COV-2 Induced Thromboinflammation

SARS-CoV-2 infection results in a more incredible release of cytokines that promote inflammation, which subsequently exacerbates chronic lung disease affecting the interstitial tissue of the lungs and progresses to viral sepsis with a notable prothrombotic state [7,8,169]. In general, viral sepsis is detected in less than 1% of all cases of viral infection [170]. According to the Sepsis-3 criteria, 20% of SARS-CoV-2-infected individuals have a severe illness, and several others may be classified as septic [171,172]. Micro thrombosis occlusions of smaller veins were found in many autopsy samples from the lungs and identified ARDS with interstitial pneumonia. Immuno-histochemical assays have also shown the presence of immune cells such as (CD (cluster of differentiation)) CD3, CD4, CD8, and other classes of CD cells in this group [173]. Patients with COVID-19 who also have comorbidities such as diabetes, obesity, and advanced age have a higher risk of venous thromboembolism, arterial thrombosis, and thrombotic microangiopathy, all of which contribute to the increased mortality reported in these patients [174]. 

**Figure 7:**
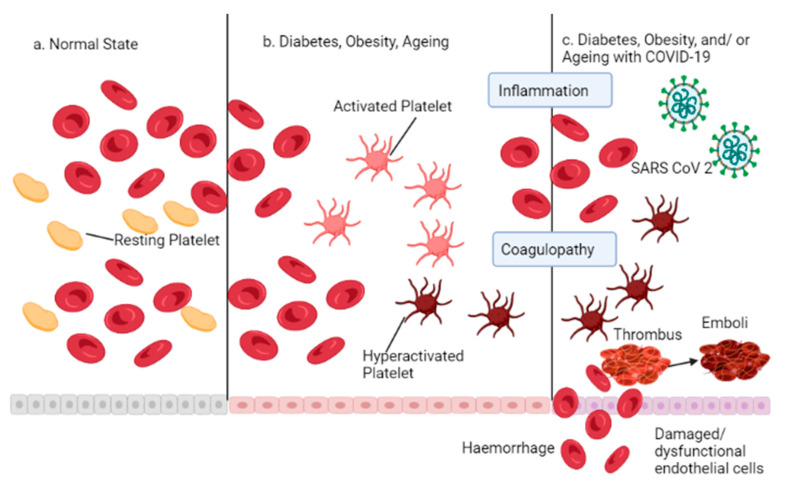
SARS-CoV-2 induced thromboinflammation.

The immunological response triggered by SARS-CoV-2 can be said to be exacerbated in the condition of “inflammageing.” This is persistent, isolated, and relatively high inflammation that occurs at old age and is characterized by a higher baseline concentration of cytokines (along with T-cell depletion, which might lead to increased mortality) [170]. Early immunosuppressant treatment should begin as soon as possible. Notably, treating the hyperinflammatory condition early will prevent the pathophysiology processes defined by immune system dysregulation [173]. Conclusively, during SARS-CoV-2 infection, early immunomodulator therapy helps avert cytokine release syndrome (CRS), sepsis-induced coagulopathy (SIC), and disseminated intravascular coagulation (DIC). [175]. The SARS-CoV-2 induced thromboinflammation is illustrated in the diagram below (Figure 7).

## 9. SARS-CoV-2 Oxidative Stress 

Oxidative stress (OS) is a physiological phenomenon in the human body caused by the imbalance between prooxidants and antioxidants, leading to an increase in the production of oxygen-reactive species (ROS) and reactive nitrogen species (RNS) [176,177,178]. The predominant ROS sources are mitochondria, NADPH oxidases (NOXs), and ROS and RNS are by-products of cellular activity [170,171,179,180]. Under normal physiological conditions, RONS are essential in various biological functions such as protein phosphorylation, activation of several transcriptional factors, apoptosis, cell signaling, thiol switches, growth factors, and regulation in inflammatory cytokines [177,180,181]. RONS’ overproduction and accumulation are harmful to the essential cell structures and functions, leading to oxidative stress [169,170]. No OS is produced in isolation during RONS production induced by viral infection [128,180,182,183]. 

Patients with severe and moderate COVID-19 infection develop respiratory distress that requires oxygen therapy that may be the leading cause of oxidative stress and ARDS. Hyperoxia leads to the production of mt-ROS that inhibits oxidative phosphorylation and lowers ATP levels causing lung tissue damage [184]. To date, it is not clear whether SARS-CoV-2 infections trigger oxidative stress in the airway epithelium [182]. The cytokine storm can also lead to cardiac oxidative stress and myocardial damage. These cardiac manifestations in infected patients occur predominantly through IL-6, TNF, and IL-1β that generate oxidative stress leading to an increase in local hypoxia, tissue injury, and redox imbalance [185]. Under oxidative stress, the resulting cytokine shock is a state of hyperinflammation accompanied by cytopenias and hyperferritinemia generated by the Fenton reaction (Fe²^+^ + H_2_O_2_ → Fe³^+^ + HO^−^ + HO^−^) and increased production of ROS [182,186]. Aging [182,186,187], male gender, black and south Asian ethnicity, low socioeconomic status, hyperglycemia, and obesity are conditions associated with enhanced oxidative stress postulated to aggravate the severity of COVID-19 infection caused by SARS-CoV-2 [182,187]. 

## 10. SARS-CoV-2 and Defective Immune Response 

### 10.1. Innate Immune Response

In SARS-CoV-2, similarly to any other viral infection, the innate immune system serves as the first line of host defense against pathogens, limiting viral entry, translation, cell division, and assembly, assisting in the identification and removal of infected cells, and accelerating the occurrence of adaptive immunity [188,189]. 

This occurs through human pattern recognition receptors (PRRs), which are vital components of the innate immune system [190,191,192], recognizing the SARS-CoV-2 virus after it enters the host’s cells [187,193], triggering inflammatory responses and programmed cell death. These receptors are also known as cytoplasmic Nod-Like Receptors 3 (NLR3), bound to and stimulated by inflammasomes [194,195,196,197,198,199,200,201]. The inflammasomes are multi-intracellular proteins that detect pathogenic microorganisms. They are secreted by the pathogen-associated molecular patterns (PAMP), damage-associated molecular patterns (DAMP), and other signaling proteins [194,195,196,197,198,199,200,201]. The circulating inflammasomes trigger the release of an active form of cytokines such as interleukin 1 beta (IL-1β) and IL-18. The process is catalyzed by the caspase-1 enzyme leading to the inflammatory response. Notably, activation of NLRP3 receptors by inflammasome may result in pyroptosis, a programmed cell death associated with hyperinflammation in macrophages and dendritic cells [194,195,196,197,198,199,200,201]. In addition, it stimulates gasdermin D, a protein cleaved by the caspase-1 enzyme leading to the worsening of hyperinflammation and eventually septic shock [194,195,196,197,198,199,200,201].

SARS-CoV-2 virus pathogenesis begins by attaching itself to the angiotensin-converting enzyme type 2 (ACE2) receptors on pneumocytes of the lung epithelium. Immediately after the virus breaches the physical barriers of pneumocytes, it will be recognized by the intracellular toll-like receptors (TLRs), which stimulate an interferon regulatory factor (IRF) (NF-kB) signaling that results in activation of NF-kβ [196,198,200,201,202,203]. Subsequently, the activated NF-kB, mitogen-activated protein kinases (MAPKs), and interferon (IFN) signal through nuclear translocation [187] stimulates cytokine production, leading to the hyperinflammatory syndrome [196,198,200,201,202,203]. Natural Killer (NK) cells are large granular lymphocytes that kill SARS-CoV-2 virus-infected cells. NK cells interact with dendritic cells, and it is hypothesized that they can directly kill virus-infected cells through degranulation, receptor-mediated apoptosis, and antibody-dependent cell-mediated cytotoxicity (ADCC) [204]. They play a significant role in lung damage among patients who have developed hyperinflammatory syndrome due to severe SARS-CoV-2 infection [196,199,200,201]. Although NK cells are not found within the lungs tissues, nevertheless, they can penetrate the lungs from the peripheral blood through the chemokine receptor-3 (CXCR3). Reports showed a considerable increase in the number of NK cells in the peripheral blood mononuclear cells detected in patients with severe COVID-19 infection compared to mild cases [196,199,200,201]. 

### 10.2. Adaptive Immune Response

The adaptive immune response begins with activating SARS-CoV-2-specific B-cell maturation and synthesis of antibodies, CD4^+^ T-cells, and CD8^+^ T-cells in response to SARS-CoV-2 infection. The role of the antibodies includes restraining the spread of the virus, suppressing the viral replication, blocking the occurrence of hyperinflammation, and cleaning the infected cells that underwent pyroptosis [196,197,198,199,200,201]. In addition, the adaptive immune system responds by inhibiting ACE2 receptors to which the SARS-CoV-2 virus attaches and invades the host’s cells. It also reacts with the virus via an autoimmune reaction following the tissue destruction [196,197,198,199,200,201]. Notably, the SARS-CoV-2 virus’ spike protein is associated with CD26 and CD147 molecules, which trigger the activation of T-cells. As a result, T-cells prevent further cellular invasion and subsequent viral replication. Overall, the activities of T-cells provoke the release of chemokines and cytokines, leading to hyperinflammatory syndrome [196,197,198,199,200,201]. The infiltration of T-cells into the tissues and cells is facilitated by the upregulation of the lung endothelial adhesion molecules causing severe lung damage and respiratory distress. The speed with which the respiratory physiology damages occur during SARS-CoV-2 infection made the mechanism by which T-cells produce hyperinflammatory syndrome unclear [196,197,198,199,200,201]. This is because several COVID-19 patients develop lymphopenia rapidly within a few days after the appearance of SARS-CoV-2 disease symptoms. However, the role of T-cells in cytokine storm is linked to tissue infiltration and cell damage at the site of infection [196,197,198,199,200,201,202,203,204,205,206]. 

### 10.3. Antibody Response

Patients infected with the SARS-CoV-2 virus expressed a humoral response by developing antibodies to tackle viral S protein used for attachment and invasion of the host’s cells. Antibodies expressed include immunoglobulin M (IgM), immunoglobulin G (IgG), and immunoglobulin A (IgA). The IgM and IgA appear within seven days of SARS-CoV-2 infection, while another antibody, immunoglobulin G (IgG), surfaced within 14 days [201,202,203,204,205,206,207]. The role of IgA as a neutralizing antibody is known, and it was detected in the bronchoalveolar lavages of people who took the COVID-19 vaccine [193,194,195,196,197,198]. As SARS-CoV-2 infection progresses, another secretory immunoglobulin A (sIgA) antibody is released. The primary function of sIgA is mucosal defense in the patient’s lungs. The SARS-CoV-2 virus attacks individuals via respiratory mucosa [199,200,201,202,203,204,205]. Despite the role of antibodies in preventing further cell invasion by the SARS-CoV-2 virus, antibodies binding to IgG Fc receptor-II positive (FcgRII+) cells, such as B-cells and macrophages, promote viral access the respiratory airways via an alternative method called canonical viral-receptor pathways [201,202,203,204,205,206,208]. Consequently, activating these receptors provokes proinflammatory cytokines leading to hyperinflammatory syndrome. This concept is called Antibody-Dependent Enhancement (ADE), another indicator of the severity of COVID-19 disease and poor treatment prognosis [201,202,203,204,205,206]. 

## 11. SARS-CoV-2 Hyperinflammation 

Patients infected with the SARS-CoV-2 virus (COVID-19) require an intact immune response to suppress the viral replication, prevent complications, and eventually survive. However, the severity and complications of COVID-19 infection that led to death are linked to the overproduction of proinflammatory cytokines known as a hyperinflammatory syndrome [17,193,195,197,202]. In a patient with severe COVID-19 disease, hyperinflammatory syndrome causes lung tissue damage similar to the features of macrophage activation syndrome (MAS) or secondary hemophagocytic lymphohistiocytosis (sHLH). However, SARS-CoV-2 associated hyperinflammatory syndrome usually produced a lesser increase in serum levels of CD25, alterations in fibrinogen, and hepatosplenomegaly making it a distinct syndrome [17,194,195,197,202]. Patients with this condition may develop respiratory collapse, thrombotic disease, and cardiac failure as the signs of disease progression and poor treatment prognosis. Hyperinflammation generally results from the active immune response to the rapidly multiplying SARS-CoV-2 virus. The immune reactions preceded this subsection, including innate immune response, adaptive immune response, and the antibody response [17,194,195,197,202]. 

### 11.1. SARS-CoV-2-Induced Hyperinflammation in Children 

Children infected with SARS-CoV-2 usually exhibit mild symptoms and, in some cases, remain asymptomatic. However, hyperinflammatory syndrome also occurs in some pediatric patients. A report indicated that about 30% of the children hospitalized due to COVID-19 might require intensive care; nonetheless, they rarely die due to severe COVID-19 infection [194,202,209]. The ability of many children to survive SARS-CoV-2 infection could be linked to the presence of a solid innate immune mechanism at the early stage of infection, which suppressed the viral replication. Additionally, children quickly acquire partial protective immunity from previous exposure to the SARS-CoV-2 virus.

Furthermore, there is reduced expression of ACE2 receptors among children, and they have less comorbidity that could weaken the quality of the lungs’ vascular endothelium and natural body immunity [194,202,209]. Despite these, a late hyperinflammatory syndrome similar to macrophage activation syndrome (MAS), toxic shock syndrome, and Kawasaki disease occurs in children. This condition is also known as a multisystem inflammatory syndrome in children (MIS-C). During this inflammatory process, there is a significant increase in serum levels of procalcitonin, C-reactive protein (CRP), ferritin, D-dimer, IL-10, and IL-6. This is also accompanied by thrombocytopenia, lymphocytopenia, and neutrophilia [183,196,202]. Ironically, children infected with COVID-19 who developed MIS-C may produce negative results in the reverse-transcriptase polymerase chain reaction (RT-PCR) COVID-19 test. However, it often reacts positively to SARS-CoV-2 serology. In general, children who developed MIS-C may be seriously ill and may need mechanical ventilation, inotropic and vasopressor support, as well as extracorporeal membrane oxygenation [194,201,209].

### 11.2. Diagnosis of SARS-CoV-2-Induced Hyperinflammation

Laboratory investigations of SARS-CoV-2-induced hyperinflammation revealed gross and microscopic pathologic parameters in a patient with severe COVID-19 infection who developed the hyperinflammatory syndrome. This revealed dysregulation of T-cells, neutrophils, macrophages/monocytes ratio, and natural killer cells [17,194,195,197,202]. In addition, other factors detected include elevated levels of systemic inflammatory biomarkers such as C-reactive protein (CRP), D-dimer, lactate dehydrogenase, and ferritin. Additionally, elevated plasma fibrinogen levels, especially in severe COVID-19-infected cases, had been a common finding [210,211]. Additionally, there is an increase in the amount of circulating proinflammatory chemokines and cytokines and a rise in the level of neutrophil-to-lymphocyte ratio [17,194,195,197,202,212]. Furthermore, SARS-CoV-2 causes a significant increase in the release of interleukins, IFN-ɣ, monocyte chemotactic peptide-1 (MCP)-1, macrophage inflammatory protein 1A (MIP)-1A, MIP-1B, GM-CSF, granulocyte-colony stimulating factor (G-CSF), TNF-α, and chemokine ligand-2 (CCL)-2 [135,137,138,140,141,142,143,149]. IL1 may be released in large quantities during hyperinflammatory syndrome and provokes cellular pyroptosis, a programmed cell death triggered by the SARS-CoV-2 virus in the epithelial cells. Overall, the presence of the above parameters in a patient with severe SARS-CoV-2 infection signifies the development of a dysregulated immune reaction known as a hyperinflammatory syndrome [17,194,196,197,199,200,201,202,212]. 

Histologic and microscopic examination of lung tissues revealed diffuse alveolar hemorrhage with edema. Interstitial and interalveolar exudates collapsed alveoli and dilated alveolar ducts, capillary congestion, desquamation of pneumocytes, and hyaline membrane formation [196,198,199,200,201]. Furthermore, large macrophages and lymphocytes were detected within the inflamed bronchioles of COVID-19 patients [128]. The macrophages above comprised infiltrated CD68^+^NP ^+^. Additionally, CD4^+^ and CD8 T-cells were found in lymphocytes extracted from the lung’s alveoli and bronchioles [128,198,200,201,202,203,213,214,215,216,217].

## 12. Therapeutic Options for SARS-CoV-2-Induced Hyperinflammation 

### 12.1. Corticosteroids

Dexamethasone and methylprednisolone are glucocorticoids used for anti-inflammatory and anti-allergy purposes. Administration of these two drugs in patients with severe COVID-19 (especially during the cytokine storm) infection may relieve endothelial injury and inflammation [17,26,199,202,203,209,212,218]. Their mechanism of action involves a decrease in the release of proinflammatory biomarkers such as soluble receptors for advanced glycation end-products (sRAGE), interleukin-6, endocan, and syndecan-1 release a decrease in endothelial injury [17,26,194,196,198,200,212,218].

### 12.2. Interleukin-6 (IL-6) Antagonists 

Tocilizumab, siltuximab, and sarilumab are recombinant humanized monoclonal antibodies with potential in treating idiopathic multicentric Castleman’s disease and cytokine-like release syndrome [198,199,200,202,212,213,214,215,216,217,218]. Their mechanism of action involves blockade of IL-6 receptors and JAK/STAT signaling pathways in the patient who developed the hyperinflammatory syndrome. They also significantly reduce the release of other proinflammatory biomarkers such as C-reactive protein, D-dimer, and ferritin [26,196,197,198,200,206,207]. These actions improve respiration and substantially raise lymphocyte count and PaO_2_/FiO_2_ ratio. They also decrease the oxygen demand and the need for mechanical ventilators, especially among the COVID-19 positive patients admitted to the intensive care unit (ICU) [26,198,199,200,202,212,213,214,215,216,217,218]. 

### 12.3. Interleuckin-1 (IL-1) Inhibitors

#### 12.3.1. Canakinumab

Canakinumab is an interleukin-1beta (IL-1β) neutralizing antibody. It can reduce hyperinflammation by binding and antagonizing inflammatory mediators such as IL-1β and IL-1α, and IL-1 decoy receptors [187,190]. Although canakinumab has proven to be helpful in COVID-19 patients suffering hyperinflammatory syndrome, targeting NLRP3 inflammasome is more effective in curbing cytokine storm [195,198].

#### 12.3.2. Anakinra 

Anakinra is a recombinant human IL-1 blocker. It also inhibits the activity of the circulating inflammasome signaling pathway in patients with severe COVID-19. Initially, it was indicated to treat rheumatoid arthritis and other autoinflammatory diseases. However, it shows promise in treating COVID-19-infected patients with hyperinflammatory syndrome [17,26,197,202]. The mechanism of action of anakinra also involves inhibition of Nod-Like Receptor 3 (NLRP3), responsible for the activity of the inflammasome signaling pathway. In severe COVID-19, anakinra produced a rapid decrease in C-reactive protein and improved oxygen supply (i.e., PaO_2_/FiO_2_ ratio) [17,26,197,202]. 

### 12.4. Janus Kinase (JAK) Inhibitors 

Baricitinib, ruxolitinib, and tofacitinib are JAK inhibitors that can suppress COVID-19-induced cytokine storm. Janus kinases are a family of enzymes, including JAK1, JAK2, JAK3, and TYK2. They are known to provoke the activity of several proinflammatory biomarkers such as interleukins, interferon, erythropoietin, and thrombopoietin growth factors [17,197,198,202]. Inhibition of these pathways may significantly relieve respiratory distress associated with hyperinflation. In addition, zanubrutinib, ibrutinib, acalabrutinib, and acalabrutinib are Bruton tyrosine kinase (BTK) inhibitors with potential in suppressing cytokine storm. BTK transmits proinflammatory biomarkers during hyperinflammation, including TLR/IL-1R, a significant signaling pathway in monocytes [17,197,198,202]. The mechanism of action of BTK inhibitors involves a decrease in the amount of circulating proinflammatory chemokines and cytokines such as IL-6, TNF-a, GM-CSF, IP-10/CXCL10, MCP-1/CCL2, MIP-1a/CCL3, and MIP-1b/CCL4. Using these drugs in a patient with severe COVID-19 who develops hyperinflammatory syndrome is associated with reduced inflammation and significant improvement in lung function [17,197,198,202]. 

### 12.5. Quercetin

Quercetin is a carbohydrate-free flavonoid, and it is the most abundant flavonoid found in vegetables and fruits. It decreases the level of NLRP3 inflammasome and adapter protein ASC, amplifies the expression of SIRT1, and activates caspase-1 [195,198]. Quercetin reduces the expression of proinflammatory cytokines, such as IL-1β, IL-18, and TNFα. It also inhibits the Janus kinase-2 and signal transducer and activator of transcription-1 (JAK2/STAT1) signaling pathway in IFN-γ-primed leukocytes. In addition, quercetin has an anti-inflammatory, analgesic, and antioxidant function, hence is suggested to be helpful in hyperinflammation caused by COVID-19 infection [195,198]. 

## 13. Conclusions

The COVID-19 pandemic has caused unprecedented damage to the global effort to provide adequate healthcare delivery services. It has continued from the first, second, and third waves, and still counting. The causative agent SARS-CoV-2 keeps evolving from α to β, gamma, and γ variants to the current Omicron and IHU, the variant of concern. With the death toll currently above five million, the devastation caused by the COVID-19 pandemic is beyond healthcare workers’ and the scientific community’s imagination. At present, no definitive cure for COVID-19 has been identified yet. Consequently, preventive measures and symptomatic treatment remain the current treatment options. Because hyperinflammatory syndrome is the major complication leading to death, targeting and managing its pathogenesis through specific cytokine storm checkpoints will go a long way in reducing mortality due to the COVID-19 pandemic.

## 14. Recommendation 

The outcome of this review suggested that more drugs from both orthodox and herbal origins that can inhibit pro-inflammatory cytokines and chemokines and prevent and treat SARS-CoV-2-induced cytokine storm should be repurposed. The medicines that showed promise against cytokine storm should be experimented with using randomized, double-blind, placebo-controlled clinical trials to generate more evidence in reducing mortality associated with COVID-19.

## Abbreviations

DC	dendritic cells
ICU	Intensive care units
MPS	mononuclear phagocyte system
mtROS	increasing mitochondrial ROS
NETs	neutrophil extracellular traps
NOXs	NADPH oxidases
RONS	reactive oxygen/nitrogen species
OS	oxidative stress
RNS	reactive nitrogen species
ROS	oxygen-reactive species
SARS-CoV	Severe Acute Respiratory Syndrome Coronavirus
